# Advanced omics approaches in liver transplant settings: current applications and future prospectives

**DOI:** 10.3389/fimmu.2025.1564248

**Published:** 2025-07-11

**Authors:** Huijuan Wang, Yifeng Zhou, Lu Yu, Zhengtao Liu, Shusen Zheng

**Affiliations:** ^1^ School of Medicine, Zhejiang Chinese Medical University, Hangzhou, China; ^2^ Department of Hepatobiliary and Pancreatic Surgery, Shulan (Hangzhou) Hospital, Shulan International Medical College, Zhejiang Shuren University, Hangzhou, China; ^3^ Key Laboratory of Artificial Organs and Computational Medicine in Zhejiang Province, Shulan International Medical College, Zhejiang Shuren University, Hangzhou, China; ^4^ National Health Commission (NHC) Key Laboratory of Combined Multi-Organ Transplantation, Key Laboratory of the Diagnosis and Treatment of Organ Transplantation, School of Medicine, Chinese Academy of Medical Sciences, First Affiliated Hospital, Zhejiang University, Hangzhou, China; ^5^ Key Laboratory of Organ Transplantation, First Affiliated Hospital, School of Medicine, Zhejiang University, Hangzhou, China; ^6^ Division of Hepatobiliary and Pancreatic Surgery, Department of Surgery, First Affiliated Hospital, School of Medicine, Zhejiang University, Hangzhou, China

**Keywords:** liver transplantation, single-cell sequencing, spatial transcriptomics, cell heterogeneity, precision medicine

## Abstract

Single-cell RNA sequencing (scRNA-seq) and spatial transcriptomics (ST), as advanced omics technologies, have addressed critical challenges in liver transplantation (LT), the most effective treatment for end-stage liver disease. This review aims to summarize the applications and future directions of scRNA-seq and ST in the context of LT. We highlight their role in uncovering immune cell heterogeneity and related injury mechanisms post-transplantation. From a clinician’s perspective, we also outline potential future developments in the application of advanced omics in LT. Specifically, we focus on key immune cells involved in LT, with an emphasis on post-transplant immune responses and ischemia-reperfusion injury (IRI), as revealed by scRNA-seq and ST. Furthermore, we underscore the importance of multi-omics approaches and dynamic omics analyses in clinical LT research. With ongoing technological advancements, the integration of cutting-edge omics technologies and artificial intelligence (AI) holds great promise for advancing precision medicine in LT. Emphasis should be placed on the value of single-cell and spatial omics technologies in improving precision therapy and clinical management for LT patients.

## 1 Introduction

Liver transplantation (LT) is widely regarded as the optimal treatment for end-stage liver disease and hepatocellular carcinoma (HCC) (1, 2). Compared to traditional surgical interventions, LT significantly improves both the quality of life and overall survival rates for patients with end-stage liver disease (3). However, several critical challenges persist in clinical practice, including donor liver shortages, ischemia-reperfusion injury (IRI), rejection, and the establishment of immune tolerance (4, 5).

Omics technologies—spanning genomics, transcriptomics, proteomics, and metabolomics—enable a comprehensive understanding of the molecular mechanisms underlying disease processes (6–8). Systems biology approach for the mechanisms underlying chronic liver disease Databases like The Cancer Genome Atlas (TCGA) released extensive omics data, offering valuable insights into the mechanisms and identifying potential translational targets for specific diseases (9). Substantial research has utilized omics approaches in LT. Our group previously reviewed the application of multi-omics data in clinical LT (10), identifying early allograft dysfunction (EAD) as a major focus of these studies. Macrosteatosis (MaS) exerted adverse effects on LT prognosis (11). Our prior metabolomics study conducted at our center identified key factors underlying MaS and graft failure (GF) (12). Subsequent research elucidated the molecular mechanisms linking MaS to graft failure, offering critical insights into donor liver quality assessment (13). These findings suggest that omics technologies provide powerful tools to investigate the genetic landscape of both donors and recipients, potentially mitigating graft failure risks and facilitating the development of therapeutic targets. However, traditional omics approaches are typically conducted at the population level, limiting their ability to capture single-cell heterogeneity or spatial organization.

In recent years, advanced omics technologies, including liquid biopsy, single-cell RNA sequencing (scRNA-seq), spatial transcriptomics (ST), single-cell methylome sequencing, single-cell multiomics, and proteome-related single-cell multimodal omics, have undergone rapid development (14–16). Among these, scRNA-seq, single-cell multiomics, and ST have emerged as particularly prominent technologies (17–19). This review focuses on the progress made in utilizing scRNA-seq and ST in LT. The rapid advancements in single-cell and spatial omics have revolutionized our understanding of cellular states and the inherent heterogeneity within biological systems (20–23). scRNA-seq provides high-resolution analyses of individual cells, offering valuable insights into cell heterogeneity and dynamic changes associated with physiological functions and human diseases (24, 25). By enabling the dissection of immune microenvironments, elucidation of injury mechanisms, and discovery of therapeutic targets, scRNA-seq has been widely applied across diverse research domains (26–28). ST is an emerging biotechnology that provided diverse insights across multiple fields (29–33). By preserving tissue architecture, ST enables the mapping of cellular and molecular distributions, enhancing our understanding of cell-cell communication, tissue structure-function relationships, and disease mechanisms (34). Together, these advanced omics technologies deepen our knowledge of cell heterogeneity, spatial localization, functionality, and interactions in the context of LT.

Although numerous studies have explored the application of advanced omics in LT (35–46), a comprehensive summary of their findings remains lacking. This review aims to synthesize prior research on advanced omics in LT and, more importantly, provide a forward-looking perspective on their clinical translation and potential contributions to the future of precision medicine in LT.

## 2 Advanced omics approaches: scRNA-seq and spatial transcriptomics

In 2009, Tang et al. (47) first reported the scRNA-seq technology, marking the beginning of rapid advancements in single-cell analysis. scRNA-seq enables the comprehensive measurement of transcript expression across large numbers of cells (48, 49) and provides an unbiased approach for identifying and characterizing distinct cell populations (50–52). This technique involves isolating and extracting RNA from individual cells, reverse-transcribing mRNA into cDNA using reverse transcription, amplifying the entire transcriptome through methods like PCR, and subsequently performing sequencing and library construction (23, 53–55). By constructing high-resolution cellular atlases, researchers can identify various cell types and subtypes, which is essential for understanding tissue functionality (56–58). In contrast, traditional next-generation sequencing (NGS), often referred to as bulk RNA-seq, analyzes RNA extracted from a bulk population of cells in a tissue, treating them as a single entity. This approach overlooks the differences between individual cells and limits analyses to the molecular level (59). scRNA-seq, on the other hand, performs analysis at the single-cell level, avoiding the homogenization effects caused by cell mixing in bulk RNA-seq. It authentically reflects cell-to-cell heterogeneity (20, 60), enabling the detailed dissection of cellular heterogeneity and providing biological insights unattainable with bulk analyses (23, 54). Recent advancements in scRNA-seq further enhance its ability to reveal novel cell types and states without bias or RNA degradation (61). In clinical LT, the application of scRNA-seq aids in identifying new cell types and subpopulations, paving the way for personalized medicine and precision single-cell therapies in clinical practice.

Spatial omics refers to methodologies used to decipher the mechanisms of biological events by identifying the relative spatial arrangement and expression of tissues, cells, and biomolecules (62). Unlike traditional omics studies, which focus solely on molecular composition (e.g., genes, proteins, and metabolites), spatial omics additionally reveal the spatial distribution and dynamic changes of these molecules at the cellular or even tissue level. The major branches of spatial omics include ST, spatial proteomics, and spatial metabolomics (34). Although spatial omics encompasses multiple approaches, most current research emphasizes ST (63). Developed in 2016 (64), ST has emerged as a major focus in biotechnology following the rise of scRNA-seq (19). ST integrates spatial resolution with gene expression analysis, enabling RNA sequencing directly on tissue sections and simultaneously detecting cellular locations and gene expression patterns (65). This capability addresses the limitation of scRNA-seq, which cannot provide information on cellular spatial distribution. By preserving the original spatial context of tissues and comprehensively measuring molecular changes, ST has revolutionized our understanding of tissue heterogeneity. It offers new avenues to explore how cellular and tissue heterogeneity contribute to functional diversity (66). While ST has provided significant insights across various fields, particularly in investigating tumor microenvironment features linked to patient prognosis (34), its application in LT clinical research remains limited.

## 3 Current application of advanced omics in LT

We reviewed and categorized the applications of scRNA-seq and ST in LT. A total of 12 studies reported the use of advanced omics technologies in LT, including 11 scRNA-seq studies and one ST study. Among these, scRNA-seq emerged as the most commonly utilized method. Most researchers favored collecting samples from graft tissues and peripheral blood. Of the studies reviewed, 8 focused on LT patient cohorts (**Table 1**), while 4 employed mouse LT models (**Table 2**). Across all studies, the 10× Genomics Chromium platform was the preferred choice. This preference likely stems from the platform’s cost-effectiveness and time efficiency compared to other scRNA-seq platforms, such as Smart-seq2. Additionally, the 10× Genomics Chromium system can process a large number of cells and even detect rare cell types or transcripts by integrating one of its advanced methodologies (67).

**Table 1:** Advanced omics study in human liver transplantation.

Author, country, publication year	Donation type	Sampling	Assays/platform	Omics	Comparison	Case number	Major findings
Yi et al., CHN, 2023 (40)	DCD	hepatic tissue	10x Genomics	*scRNA-seq*	pre-LT vs. post-LT	3 vs. 3	LT changed the immune microenvironment of the donor's liver.
Fang et al. CHN, 2023 (42)	No description	peripheral blood	10x Genomics	*scRNA-seq*	health vs. post-LT	1 vs. 4	CD8+ NKG2A+ natural killer T cells are disrupted after liver transplantation and immunosuppressive therapy.
Li et al., CHN, 2022 (43)	DCD or LRD	hepatic tissue, peripheral blood	10x Genomics	*1.scRNA-seq* *2.bulk RNA-seq*	health vs. post-LT	3 vs. 4	1. There was heterogeneity of different T cells in the liver after LT. 2.kupffer cell subsets had different changing trends; 3.LDLR can be used as a new marker to prevent LT rejection; 4. CD4+CD8+FOXP3+ T cell subsets were detected in human LT for the first time; 5.LDLR+ MDSC and CTLA4+CD8+ T cells interact through the TIGIT-NECTIN2 signaling pathway
Li et al., CHN, 2024 (38)	DCD or LRD	hepatic tissue, peripheral blood	10x Genomics	*1.scRNA-seq* *2.bulk RNA-seq*	ACR vs. no ACR	9 VS. 8	1. The proportion of CD8+TRM in rejection increased significantly, with a unique expression profile; 2. Interactions with TRMS, such as kupffer cells are particularly active during rejection.
Zhang et al., CHN, 2024 (36)	DCD or LRD	hepatic tissue, peripheral blood	10x Genomics	*scRNA-seq*	ACR vs. no ACR	22 vs. 28	1.FLT3+ dendritic cells decreased in peripheral blood and liver tissue of liver transplantation patients, while regulatory T cells increased; 2.FLT3+ dendritic cells inhibit immune rejection by interacting with regulatory T cells.
Wang et al., CHN, 2024 (37)	DCD or LD	peripheral blood	10x Genomics	*1.scRNA-seq* *2.bulk RNA-seq*	health vs. post-LT	6 vs. 5	1. Immune recovery after LT can be divided into four stages; 2. Inflammatory NK cell subsets, CD14+RNASE2+ monocytes and FOS-expressing monocytes were used as predictors of transplant rejection.
Wang et al., CHN, 2021 (45)	BCD	hepatic tissue	10x Genomics	*1.scRNA-seq* *2.bulk RNA-seq*	PP vs. EP vs. PR	1 vs. 1 vs.1	The expression of TNFAIP3 was significantly up-regulated in Kupffer cell population after reperfusion, which may have a protective effect on ischemia-reperfusion injury.
Zhang et al., CHN, 2024 (35)	No description	hepatic tissue, peripheral blood	10x Genomics	*1.scRNA-seq* *2.bulk RNA-seq*	pre-LT vs. post-LT	30 vs. 30	MDSCs are recruited in liver ischemia-reperfusion injury via CXCL17-GPR35 signaling, inhibiting M1 macrophage polarization and reducing liver damage.

DCD, donor liver after cardiac death; LD, living donor; DBD, liver donor after brain death; PP, pre-procurement; EP, the end of preservation; PR, post-reperfusion; scRNA-seq, Single-cell sequencing.

**Table 2:** Advanced omics study in animal liver transplantation model.

Author, country, publication year	Intervention	Sampling	Platform	Omics	Comparison	Number	Major findings
Li et al., CHN, 2023 (41)	1.IS 2.IS+MSCs	hepatic tissue	10x Genomics	*scRNA-seq*	Syn vs. Allo+IS vs. Allo+IS+MSCs	8 vs.8	1. Immunosuppressant treatment alone or combined with mesenchymal stem cells changed the intrahepatic cell landscape and the gene expression pattern of immune cells after LT. 2. Mesenchymal stem cells may be involved in the differentiation of T cells, classical monocytes and non-classical monocytes.
Adra et al., USA, 2024 (39)	1.NEVLP 2.NEVLP-cyt	hepatic tissue	10x Genomics	*scRNA-seq*	SCS vs. NEVLP vs. NEVLP-cyt	1vs.1vs.1	1.NEVLP was associated with downregulation of multiple gene enrichment pathways; 2. The addition of anti-inflammatory cytokines can further down-regulate the genes in TRM T cells, most of which are pro-inflammatory genes
Yang et al., CHN, 2021 (44)	high-fat diet	hepatic tissue	10x Genomics	*scRNA-seq*	CDL vs. FDL	3 vs. 3	After fatty LT, Kupfer cells and DCs exhibit different immunophenotypes associated with ischemia-reperfusion injury.
Xin et al., CHN, 2023 (46)	1. I/R 2.celastrol	hepatic tissue	GeoMx DSP WTA	*ST*	Health vs. IRI vs. IRI+ celastrol	4~10 vs.4~10 vs. 4~10	1. Hepatic ischemia-reperfusion injury mainly affects the central area of the hepatic lobule. 2.Celastrol alleviates hepatic ischemia-reperfusion injury by activating HIF1α signaling pathway.

Syn, syngeneic; Allo, allogenic; IS, immunosuppressant; MSCs, Mesenchymal stem cells; NEVLP, Isolated liver perfusion at normal temperature; NEVLP-cyt, NEVLP+ Anti-inflammatory cytokines; SCS, Static cold storage; CDL, Control the donor liver; FDL, Fat donor liver; I/R, ischemia-reperfusion; IRI, ischemia-reperfusion injury; scRNA-seq, Single cell sequencing; ST, Spatial transcriptomics.

## 4 scRNA-seq reveals LT immune cell heterogeneity

Following LT, the interaction between donor-derived resident immune cells and recipient immune cells leads to the establishment of a novel immune microenvironment (68). Immune microenvironment is a complex ecosystem comprising various immune cells, cytokines, and signaling molecules, all of which interact to modulate the progression and outcome of patients with chronic liver diseases (69). As the immune microenvironment evolves, changes occur in the quantity, proportions, phenotypes, and functions of immune cell populations, ultimately contributing to graft injury and its progression after LT (70). Additionally, during IRI in LT, various cell types participate in the injury process through distinct mechanisms (71, 72). Significant advancements in scRNA-seq research within the LT field have deepened our understanding of the liver ecosystem and immune microenvironment. This has enabled scientists to uncover the complexities and cellular heterogeneity of the liver during transplantation (23, 73). The analysis of scRNA-seq data typically involves quality control, batch effect correction, normalization, data imputation, dimensionality reduction, subsequent expression analyses, and cell subgroup identification (74). Using scRNA-seq to analyze intrahepatic immune cells provides detailed insights into the heterogeneity of different immune cell populations involved in LT immune rejection and IRI (75). Below, we summarize the heterogeneity and changes in T cells, B cells/plasma cells, NK/NKT cells, dendritic cells (DCs), Kupffer cells, monocytes, and myeloid-derived suppressor cells (MDSCs) in the context of LT. Specific subpopulations and their changes are detailed in the corresponding tables (**Tables 3**–**9**).

**Table 3:** Subpopulations and changes in T cells.

Author, country, publication year	Subgroup Identification	Quantitative Changes(post-LT)
Zhang et al., CHN, 2024 (36)	*CD4+ T: Tregs, Th1, Th2, Th17;* *CD8+ T: CTLs, Tmem*	Tregs increases
Fang et al., CHN, 2023 (42)	*naïve CD8+ T cells, naïve CD4+ T cells, CD4+ Tem, γδ-T cells, Teff*	No
Li et al., CHN, 2022 (43)	*CD4+T: CCR6+CD4+T, IL7R+CD4+T, CCR7+CD4+T, CCR6+CD4+T;* *CD8+T: CTLA4+CD8+T, MKI67+CD8+T;* *CCL3-CD4-CD8-T*	CCR6+CD4+T、CTLA4+CD8+T cells、MKI67+CD8+T cells increase
Li et al., CHN, 2024 (38)	*CD8+ T: TRM, Tc, TEM, TEX, MAIT, DNT*	CD8+ TRMs significantly increase rejection
Yi et al., CHN, 2023 (40)	*CD4+ T: Tem, Naïve T cells, MAIT, T cycling cells;* *CD8+ T: MAIT, Tem, Naïve T cells, Temra*	CD4+ Tem, CD8+ Tem, Temra T cells; MAIT decrease
Wang et al., CHN, 2024 (37)	*CD4+T: naïve CD4+ T cells, central memory CD4+ T cells, Tregs;* *CD8+ T: naïve CD8+ T cells, anergic CD8+ T cells, Tmem, CTLs*	Stage 1: T cells decrease; Stage 2-4: T cells increase
Li et al., CHN, 2023 (41)	*CD8+ T: Teff, Tex, Th, Tregs, naïve T cells*	Allo+IS: Teff and Th increase; Tregs and naïve T cells decrease Allo+IS+MSCs: Tregs increase; Teff and Th decrease
Yang et al., CHN, 2021 (44)	*CXCR3+ CD8+ T cells, CCR7+ CD8+ T cells, Mki67+ CD8+ T cells*	FDL: CCR7+ CD8+ T cells increases
D.A. Adra et al., USA, 2024 (39)	*TRM, TEM, TCM*	NEVLP-cyt: TRM decrease
Wang et al., CHN, 2021 (45)	*CCR7+ CD8+ Tem, CCL20+ CD8+ MAIT, IL7R+ CD4+ T, TRBV9+ CD8+ Teff, TRDV2+ γδ T*	PR: IL7R+ CD4+ T, TRBV9+ CD8+ Tef, CCR7+ CD8+ Tem increases

Tregs, regulatory T cells; CTLs, Cytotoxic T cells; Temra, Terminally differentiated T cells; Tem, Effector memory T cells; Teff, effector T cells; TRM, Tissue resident memory T cells; Tc, Cytotoxic T cells;Tex, exhausted T cells; Th, helper T cells; MAIT, Mucosal associated invariant T cells; DNT, Double negative T cells; Allo, allogenic; IS, immunosuppressant; MSCs, Mesenchymal stem cells; FDL, Fat donor liver; NEVLP-cyt, NEVLP+ Anti-inflammatory cytokines; PR: post-reperfusion.

**Table 4:** Subpopulations and changes in B cells.

Author, country, publication year	Subgroup Identification	Quantitative Changes(post-LT)
Fang et al., CHN, 2023 (42)	*pre-B cells, naïve B cells*	No
Li et al., CHN, 2022 (43)	*RASSF6+B; ZBTB16+B; FCRL3+B; IL6+B; IGHG+B; HRK+B; IL32+B; IGHA2+B*	IL4R+B cells、NEIL1+B increase; AIM2+B cells、IGLL5+B cells decrease
Yi et al., CHN, 2023 (40)	*Naive B cells; Memory B cells; Plasma cells*	Memory B cells increase; Plasma cells decrease
Li et al., CHN, 2023 (41)	*Memory B cells, plasma cells*	Allo+IS: B cells increase; Allo+IS+MSCs: B cells decrease
Wang et al., CHN, 2021 (45)	*TCL1A+ B cell, GPR183+ B cell, MT2A+ B cell*	No

Allo, allogenic; IS, immunosuppressant; MSCs, Mesenchymal stem cells.

**Table 5:** Subpopulations and changes in NK/NKT cells.

Author, country, publication year	Subgroup Identification	Quantitative Changes(post-LT)
Fang et al., CHN, 2023 (42)	*CD8+ NKT cells (E1, E2, C5)*	CD8+ NKT cells increase, then decrease
Li et al., CHN, 2022 (43)	*XCL1+NK; FGFBP2+NK*	FGFBP2+NK cells increase
Yi et al., CHN, 2023 (40)	*NK cell cycling cells; KLRC1+ NK cells; CD16+ KLRC1+ NK; CD16+ NKT cells; CD16+ NK cells*	CD16+ NKT cells increase; KLRC1+ NK cells decrease
Wang et al., CHN, 2024 (37)	*CD56brightCD16− NK cells;* *CD56dimCD16+ NK cells*	Stage 1: NK cells decrease; Stage 2-4: NK cells T cells
Li et al., CHN, 2023 (41)	No	Allo+IS: NKT cells increase; Allo+IS+MSCs: NKT cells decrease
Wang et al., CHN, 2021 (45)	*GNLY+ NK, PTGDS+ NK, XCL2+ NK, XCL1+ NK; STMN1+ T-cycling, STMN1+ NK-cycling*	PR: PTGDS+ NK increase

Allo, allogenic; IS, immunosuppressant; MSCs, Mesenchymal stem cells; PR, post-reperfusion.

**Table 6:** Subpopulations and changes in dendritic cells.

Author, country, publication year	Subgroup Identification	Quantitative Changes(post-LT)
Zhang et al., CHN, 2024 (36)	*pDC, mature DC, CD209+cDC2, CD1c+DC, CCL5+cDC, FLT3+cDC1*	FLT3+DCs decreases
Li et al., CHN, 2022 (43)	*CD1C+DC; SDS+DC; CADM+DC; CD141+DC*	DC cells increase
Yi et al., CHN, 2023 (40)	*cDC; pDCs*	No
Li et al., CHN, 2023 (41)	*pDCs; cDC1; cDC2*	Allo+IS+MSCs: pDCs increase
Yang et al., CHN, 2021 (44)	*XCR1+ DCs*	FDL: XCR1+ DCs increase

pDCs, Plasmacytoid dendritic cells; cDC, conventional dendritic cells; Allo, allogenic; IS, immunosuppressant; MSCs, Mesenchymal stem cells; FDL, Fat donor liver.

**Table 7:** Subpopulations and changes in kupffer cells.

Author, country, publication year	Subgroup Identification	Quantitative Changes(post-LT)
Li et al., CHN, 2022 (43)	*CD163+Kupffer; APOE+Kupffer; GZMA+Kupffer*	FOLR3+Kupffer cells increase; CD163+Kupffer cells、GZMA+Kupffer cells decrease
Yi et al., CHN, 2023 (40)	*Classical Kupffer cells;Inflammatory Kupffer cells*	Inflammatory Kupffer cells increase; Classical Kupffer cells decrease
Li et al., CHN, 2023 (41)	No	Allo+IS: Kupffer cells increase; Allo+IS+MSCs: Kupffer cells decrease
Yang et al., CHN, 2021 (44)	*CSF3+ Kupffer cells*	FDL: CSF3+ Kupffer cells increase
Wang et al., CHN, 2021 (45)	*C1QC+ Kupffer cells, IL1B+ Kupffer cells*	PR: Kupffer cells decrease

Allo, allogenic; IS, immunosuppressant; MSCs, Mesenchymal stem cells; FDL, Fat donor liver; PR, post-reperfusion.

**Table 8:** Subpopulations and changes in monocytes.

Author, country, publication year	Subgroup Identification	Quantitative Changes(post-LT)
Yi et al., CHN, 2023 (40)	*CD14+ Monocytes;CD16+ Monocytes*	Monocytes increase
Wang et al., CHN, 2024 (37)	*CD14+ monocytes; CD14+RNASE2+ monocytes, FOS-expressing monocytes*	Stage 1: CD14+ monocytes, CD14+RNASE2+ monocytes increase but then decrease Stage 2: CD14+CD16+ monocytes increase
Li et al., CHN, 2023 (41)	*Classical monocytes; non-classical monocytes*	Allo+IS: Classical monocytes increase; Allo+IS+MSCs: non-classical monocytes increase
Yang et al., CHN, 2021 (44)	*Mono1, Mono2, Mono3*	No
Wang et al., CHN, 2021 (45)	*VCAN+ TMo, S100A8+ TMo*	PR: monocytes increase
Zhang et al., CHN, 2024 (35)	*CD14+ monocytes;VCAN+ monocytes*	CD14+ monocytes increase

Allo, allogenic; IS, immunosuppressant; MSCs, Mesenchymal stem cells; PR, post-reperfusion.

**Table 9:** Subpopulations and changes in myeloid-derived suppressor cells.

Author, country, publication year	Subgroup Identification	Quantitative Changes(post-LT)
Li et al.,CHN, 2022 (43)	*LDLR+MDSC*	LDLR+MDSC increases
Zhang et al., CHN, 2024 (35)	*Mdsc-like cells:* *S100A9+ monocytes;S100A12+granulocyte*	S100A9+ monocytes,S100A12+granulocyte increase

MDSC, Myeloid suppressor cells.

### 4.1 T cells

T cells are critical components of the adaptive immune response. In LT, T cells can be categorized into different subtypes based on their functions, with specific subtypes directly attacking “foreign” hepatocytes, ultimately leading to acute rejection (76). All scRNA-seq studies on LT (35–45) annotated T cells, though the specific subtypes identified varied across studies. Notably, two studies (38, 39) highlighted the significance of tissue-resident memory T cells (TRMs) in LT. Li et al. (38) discovered a dual phenotypic role of CD4+ TRMs in LT rejection cases, with CD8+ TRMs playing a dominant role during LT. Adra et al. (39) used a mouse IRI model to study the effects of normothermic ex vivo liver perfusion (NEVLP) on TRMs. They observed that the proportion of TRMs among total T cells remained similar across mouse livers subjected to different mechanical perfusion conditions. However, in NEVLP-treated livers supplemented with IL-10 and TGF-β, both the total T cell population and TRMs were significantly reduced. Additionally, Li et al. (43) were the first to detect CD4+CD8+FOXP3+ T cells in human LT, providing new insights into T cell subtypes in the context of transplantation. (**Table 3**)

### 4.2 B cells/plasma cells

B cells act as antigen-presenting cells (APCs) in LT, recognizing and binding foreign antigens through B cell receptors on their cell membranes and presenting them to T cells, thereby activating a specific immune response (77). Three studies (40, 43, 45) analyzed and annotated B cell/plasma cell populations. However, their findings appear to differ across studies. Wang et al. (45) reported that the expression profile of B cell/plasma cell populations remained largely unchanged after IRI. In contrast, Li et al. (43) identified two B cell subpopulations that were significantly enriched in LT patient tissues compared to healthy tissues. Additionally, Yi et al. (40) found that the proportion of memory B cell subpopulations in the liver increased significantly after LT, while the proportion of plasma cells decreased (**Table 4**).

### 4.3 NK/NKT cells

NK cells are essential innate immune cells capable of recognizing and eliminating tumor cells and virally infected cells. In LT, NK cell activity is closely associated with graft rejection (76). NKT cells, a hybrid of T cells and NK cells, play a regulatory role in immune responses. In LT, NKT cells influence the activity of other immune cells by secreting cytokines, thus playing a critical role in regulating immune tolerance and anti-rejection mechanisms (78). Four studies (37, 40, 42, 43) have focused on analyzing different NK/NKT cell subpopulations. Li et al. (43) found an increased proportion of FGFBP2+ NK cells in the liver after LT. Yi et al. (40) reported a sharp increase in CD16+ NKT cells and a decrease in KLRC+ NKT cells in post-LT liver tissues. In contrast, Wang et al. (37) observed that the proportion of NK subpopulations decreased in both acute cellular rejection (ACR) and non-ACR LT patients post-transplant, though the functional changes of NK subpopulations varied significantly between these groups. Notably, two studies reported changes in NK/NKT cell subpopulations following immunosuppressive therapy in LT patients. Wang et al. (37) observed a reduction in inflammatory NK cells after high-dose methylprednisolone (MePDN) treatment in ACR patients. Fang et al. (42) identified a significant decrease in CD8+ NKG2A+ NKT cells specifically expressing KLRC1 after immunosuppressive therapy (**Table 5**).

### 4.4 Dendritic cells (DCs)

DCs are critical antigen-presenting cells that activate T cells and initiate immune responses. DCs associated with LT play a pivotal role in regulating allogeneic responses, particularly through their ability to induce diverse phenotypes and functional states that can either enhance or suppress immune responses (79). In LT, DCs influence graft outcomes by modulating immune tolerance and promoting T cell activation. Studies have shown that DC dysfunction can lead to increased graft rejection, highlighting their indispensable role in immune regulation (43, 78). Two studies (36, 44) have specifically analyzed changes in DC subpopulations in LT. Yang et al. (44) identified a novel DC subpopulation (XCR1+ DCs) and predicted that these cells could promote T cell proliferation. Zhang et al. (36) reported a significant reduction in FLT3+ DCs in both peripheral blood and liver tissues after LT. Furthermore, they identified a negative correlation between the proportion of FLT3+ DCs and regulatory T cells (Tregs) in post-LT liver tissues (**Table 6**).

### 4.5 Kupffer cells

Kupffer cells are macrophages residing in the liver, which can not only act as APCs but also induce the immune response of T cells by expressing major histocompatibility complex (MHC) class II molecules. But also play a key role in regulating inflammatory response and maintaining immune tolerance (80–83). Five studies (40, 41, 43–45) have conducted a detailed analysis of the heterogeneity and changes of kupffer cells in LT. Among them, three studies (40, 43, 45) emphasized that kupffer cells possess both pro-inflammatory and anti-inflammatory dual effects in LT. Li et al. (41) proposed that the differentially expressed genes of kupffer cell subpopulations could serve as immune response biomarkers for patients undergoing LT and immunosuppressive therapy. It is noteworthy that Yang et al. (44) discovered a new pro-inflammatory kupffer cell subpopulation (CSF3+ kupffer cells) and suggested that this subpopulation may be a key subset involved in initiating the pro-inflammatory response during IRI (**Table 7**).

### 4.6 Monocytes

In LT, monocytes can influence the intensity and nature of rejection through their antigen-presenting function. Studies have shown that high levels of monocytes may be associated with strong rejection, while low levels may be associated with immune tolerance (84). Three studies (37, 40, 41) have focused on the heterogeneity and changes of monocytes in LT. Yi et al. (40) found that CD14+ and CD16+ monocytes in donor livers expressed the activation of multiple pro-inflammatory pathways. Li et al. (41) discovered that the combination of immunosuppressants and mesenchymal stem cells (MSCs) could significantly reduce the proportion of classical monocytes while increasing the proportion of non-classical monocytes. This indicates that the combination of immunosuppressants and MSCs may affect allogeneic immune responses in transplantation by acting on classical and non-classical monocytes. Notably, Wang et al. (37) proposed that the newly identified CD14+RNASE2+ monocytes and monocyte subtypes expressing FOS could serve as predictive indicators of ACR in LT (**Table 8**).

### 4.7 MDSC

MDSC is a heterogeneous population of bone marrow cells with unique immunosuppressive functions, which were initially studied in tumors (85). Research has shown that MDSCs can suppress T lymphocytes and limit innate immune responses in infections, inflammatory diseases, and ischemic diseases (86–89). Among all the studies, two studies (35, 43) focused on the heterogeneity and changes of MDSCs in LT. Li et al. (43) found that the proportion of LDLR+ MDSCs significantly increased in LT tissues, especially in rejection tissues. Zhang et al. (35) established a mouse model of liver IRI and found that peripheral MDSCs were recruited to the liver after IRI. During liver IRI, MDSCs could enhance the suppression of intrahepatic inflammation and tissue damage (**Table 9**).

## 5 Analysis of LT-related mechanisms using scRNA-seq

LT rejection reactions mainly include hyperacute rejection, acute T cell-mediated rejection (TCMR), acute antibody-mediated rejection (AMR), and chronic rejection (90). TCMR is the most common form of allograft injury, primarily occurring within the first 3 months after LT, and most frequently within the first 6 weeks (91, 92). Severe rejection reactions can lead to transplant failure and may even necessitate re-transplantation; therefore, timely identification and management of rejection reactions are crucial for improving transplant success rates.IRI is a major complication of various liver surgeries, especially in LT. IRI refers to the damage caused by the interruption of blood flow (ischemia) and the subsequent restoration of blood flow (reperfusion) during organ transplantation. IRI is a progressive process that can ultimately lead to acute liver dysfunction and even allograft rejection in recipients (93). During reperfusion, oxidative stress causes hepatocyte death, followed by the release of damage-associated molecular patterns that activate hepatic macrophages and trigger innate immune responses (94, 95), ultimately resulting in dysfunction of the transplanted liver. Both LT rejection reactions and IRI are significant barriers to reducing transplant success rates and long-term survival rates. Therefore, understanding the mechanisms related to LT rejection reactions and IRI is essential.

### 5.1 Immune Responses in LT

**Figure 1:**
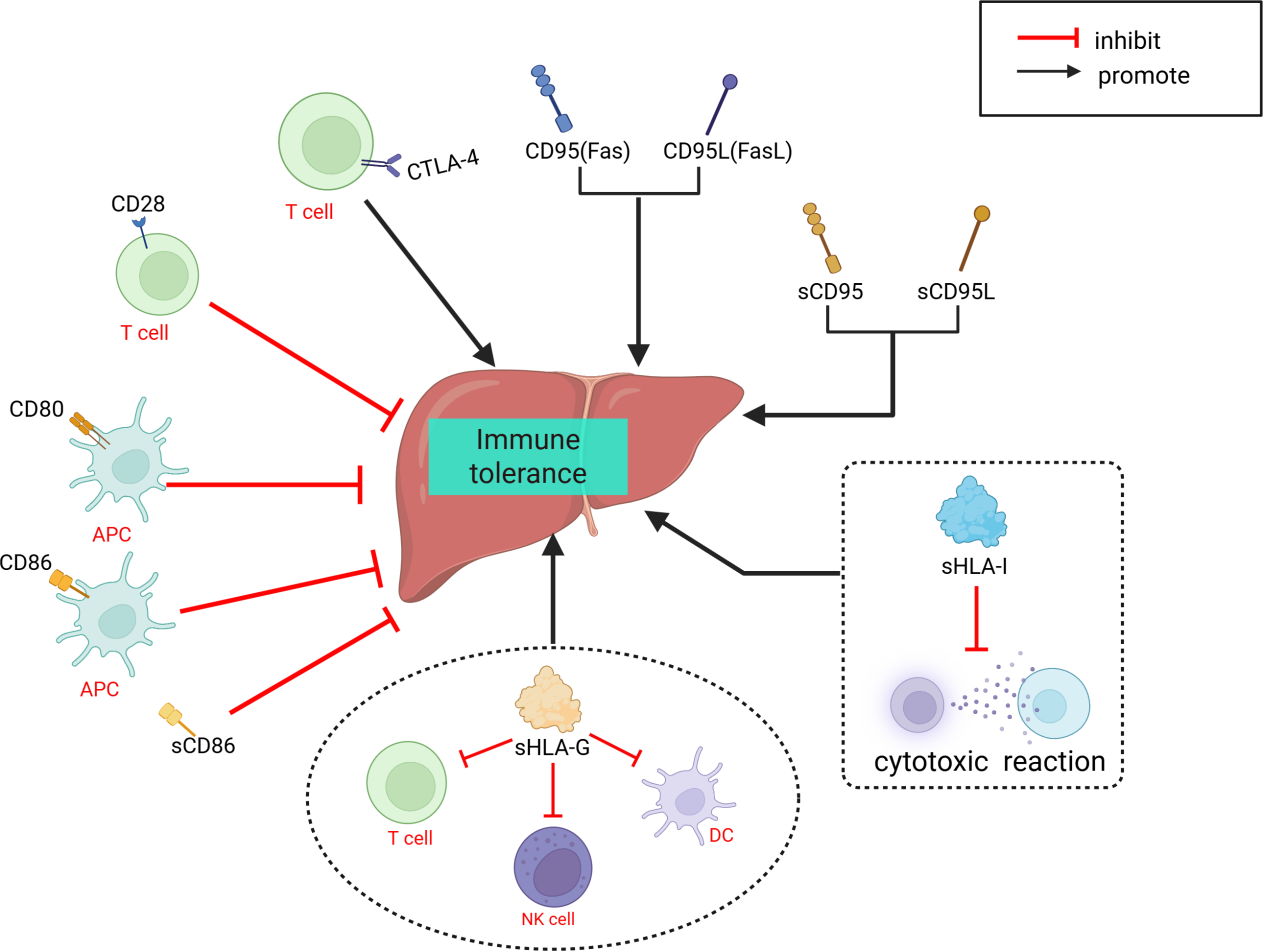
Role of various immune molecules in liver transplantation immune tolerance. APC, antigen-presenting cell; CTLA-4, Cytotoxic T-lymphocyte-associated protein 4; DC, dendritic cell; Fas, Factor associated suicide; FasL, Factor associated suicide ligandHLA-G, Human leukocyte antigen G; HLA-I, Human leukocyte antigen I.

**Figure 2:**
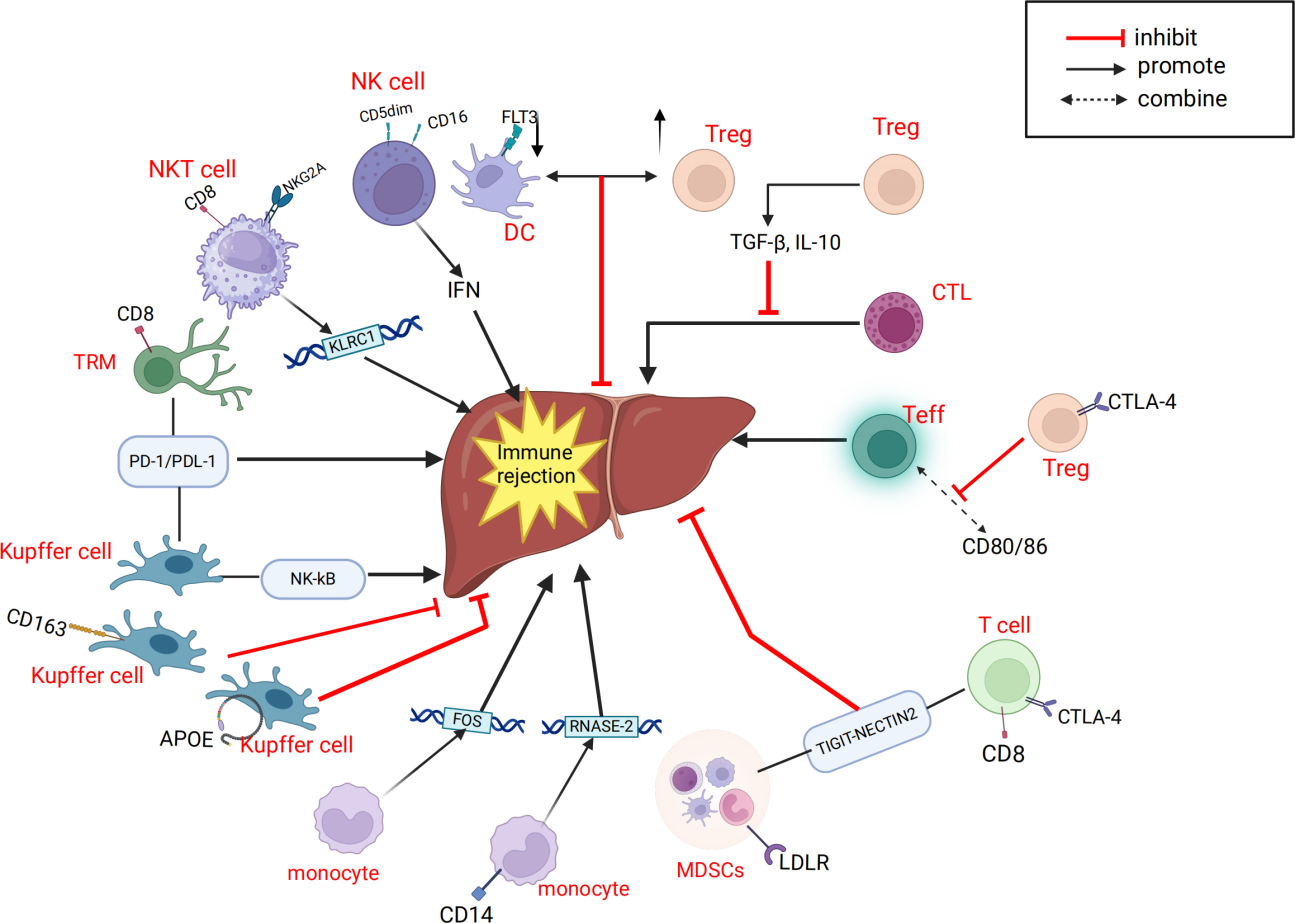
Related cells and molecules that influence liver transplantation rejection. APOE, Apolipoprotein E; CTL, Cytotoxic T cell; CTLA-4, Cytotoxic T-lymphocyte-associated protein 4; DC, dendritic cell; IRI, ischemia-reperfusion injury; FLT3, Fms-like tyrosine kinase 3; FOS, Fos Proto-Oncogene, AP-1 Transcription Factor Subunit; KLRC1, killer cell lectin like receptor C1; LDLR, Low density lipoprotein receptor; MDSC, myeloid-derived suppressor cell; NK, natural killer; NKT, natural killer; NKG2A, natural killer cell lectin-like receptor subfamily C member 1; NECTIN2, Nectin Cell Adhesion Molecule 2; RNASE-2,Ribonuclease A Family Member 2; TRM, memory T cell; Treg, regulatory T cell; Teff, effector T cell; TIGIT, T cell immunoreceptor with Ig and ITIM domains; PD-1, Programmed Death-1; PDL-1, Programmed Cell Death-Ligand 1.

The involvement of immune cells in LT rejection or immune tolerance is complex. Compared to other solid organ transplants, immune regulation within liver allografts exhibits distinct characteristics. As summarized in the review by Muro et al. (96), the liver contributes significantly to host defense by synthesizing various immunomodulatory molecules. Multiple well-characterized costimulatory molecules and receptors—such as CD28, CD95, CD95L, CTLA-4, CD80, and CD86 (97–100)—as well as soluble immunoregulatory factors including sHLA-I, sHLA-G, sCD86, sCD95, and sCD95L (101, 102), have been shown to play pivotal roles in shaping LT immune responses, particularly in the induction and maintenance of immune tolerance (**Figure 1**). In addition, the unique immunological microenvironment of the liver allograft further contributes to these regulatory processes, different immune cells play distinct roles in the immune response process and contribute differently to the establishment of immune rejection or tolerance (103, 104). Researchers have proposed various perspectives on the immune responses in LT. Fang et al. (42) suggested that CD8+NKG2A+NKT cells specifically expressing KLRC1 may be involved in immune rejection reactions, and dynamic monitoring of these cells may aid in detecting the development of long-term immune tolerance after LT. Li et al. (43) identified LDLR as a novel marker for activating MDSCs and proposed that the interaction between LDLR+MDSCs and CTLA4+CD8+ T cells through the TIGIT-NECTIN2 signaling pathway can suppress LT rejection reactions. In a subsequent study, Li et al. (38) found that CD8+TRM cells have a significant advantage in LT rejection reactions and emphasized their potential involvement in important signaling pathways that maintain and develop rejection reactions. Zhang et al. (36) reported a negative correlation between FLT3+ DCs and Tregs in the liver after LT, and proposed that FLT3+ DCs may regulate immune responses in LT by mediating Treg dynamics. Li et al. (41) discovered in a mouse LT model that the functions of LT immune cells can be altered by immunosuppressants and MSCs, and suggested utilizing the immunosuppressive effects of MSCs on immune cells to modulate early inflammatory responses in LT. Wang et al. (37) elucidated the longitudinal evolution of immune cells during LT recovery under tacrolimus-based immunosuppressive therapy and provided a four-stage framework that aids in the clinical management of LT patients. We have summarized the aforementioned immune response mechanisms related to LT (**Figure 2**).

### 5.2 IRI and organ protection

**Figure 3:**
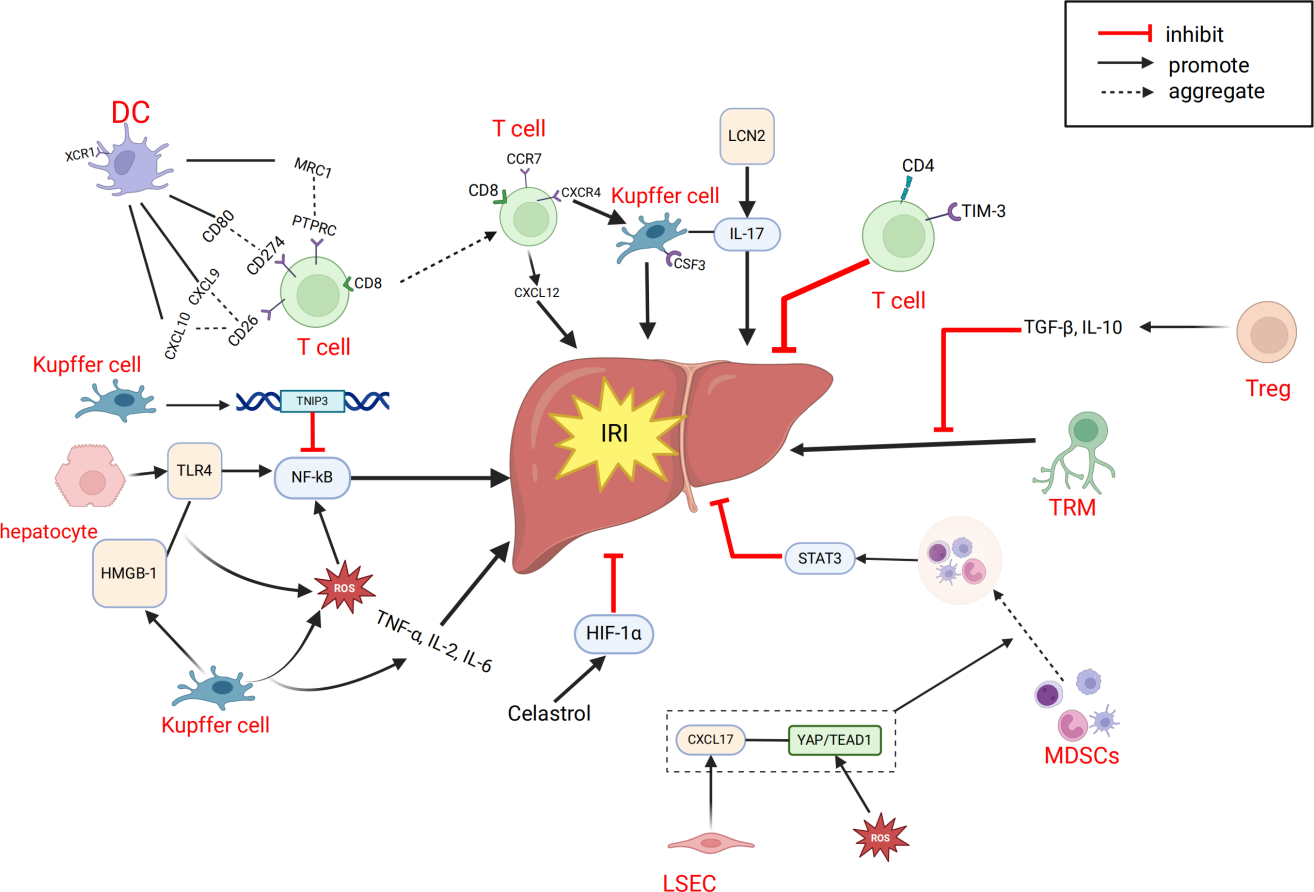
Related cells and molecules affecting hepatic ischemia-reperfusion injury. CXCL, C-X-C motif chemokine ligand; CCR7,CC-chemokine receptor 7; CXCR4, C-X-C chemokine receptor type 4; CSF3, colony stimulating factor 3; DC, dendritic cell; IRI, ischemia-reperfusion injury; HMGB-1, High Mobility Group protein B1; LCN2, Lipocalin-2; MRC1, Mannose Receptor C-type 1; MDSC, myeloid-derived suppressor cell; PTPRC,Protein Tyrosine Phosphatase, Receptor Type, C; ROS, reactive oxygen species; TRM, memory T cell; Treg, regulatory T cell; TNIP3, TNFAIP3 interacting protein 3; TLR4, Toll-like receptor 4; TIM-3, T-cell immunoglobulin and mucin domain 3.

Systematic and comprehensive analysis of the single-cell transcriptome of intrahepatic cells during LT can help us gain a deeper understanding of the mechanisms related to IRI. Excessive inflammation caused by Kupffer cells is a key mechanism leading to pathological damage in IRI. After reperfusion, accumulated endogenous damage-associated molecular patterns and pathogen-associated molecular patterns are released into the liver, activating Kupffer cells and inducing the production of reactive oxygen species (ROS), TNF-α, IL-1β, and other pro-inflammatory cytokines, forming a positive feedback loop (105, 106). Wang et al. (45) found that TNFAIP3 interacting protein 3 (TNIP3) is highly expressed in kupffer cell clusters during IRI and proposed that TNIP3 may be one of the protective mechanisms for alleviating graft damage by inhibiting the activation of the NF-κB pathway, with the TNIP3 gene potentially serving as a therapeutic target for IRI. Fatty livers may be more susceptible to IRI, which is a major cause of liver injury and is unfavorable for liver regeneration (107, 108). Yang et al. (44) analyzed two groups of rat LT models, normal donor livers (CDL) and fatty donor livers (FDL), and identified two new cell subtypes, CSF3+kupffer cells and XCR1+ DCs. They proposed that CSF3+kupffer cells may be a key subset of kupffer cells initiating pro-inflammatory responses in fatty donor liver IRI, and that IL-17 signaling in CSF3+ kupffer cells is a potential regulatory axis for cellular response to hypoxic IRI. LCN2 is one of the upregulated genes involved in the IL-17 signaling pathway and participates in many inflammation-related pathways, potentially serving as a therapeutic target for preventing fatty liver graft damage in LT. Some studies have shown that the ischemic/hypoxic environment in IRI of solid tumors or organs recruits peripheral MDSCs and exerts immunosuppressive functions in cancer and inflammation (87, 109–111). Zhang et al. (35) proposed that MDSCs also have immunosuppressive functions within the liver. During liver IRI, the YAP/TEAD1 signaling pathway upregulates CXCL17, which recruits MDSCs. Subsequently, MDSCs upregulate the STAT3 signaling pathway, enhancing their immunosuppressive functions and thereby alleviating graft IRI. NEVLP is a transplant liver preservation strategy with advantages over static cold storage (SCS) (112, 113). Adra et al. (39) found that in mouse livers subjected to NEVLP with the addition of anti-inflammatory factors (IL-10 and TGF-β), both total T cells and tissue-resident memory T cells (TRM) were significantly reduced. The addition of anti-inflammatory factors during mechanical perfusion and preservation of donor livers may potentially reduce IRI-induced liver damage. We have also summarized the important cellular and molecular mechanisms related to IRI in LT, as mentioned above (**Figure 3**).

## 6 Application of ST in LT

The liver has a complex structure with distinct functional zoning, based on the liver lobule as the fundamental unit, which is divided into different oxygen-supplied regions, each with specific functions. There have already been some studies on ST in the liver or liver diseases. For example, some studies have shown that liver injury patterns are region-dependent (114–118). Other research has used ST to analyze the regional-specific gene expression patterns of the liver under homeostasis, identifying differentially expressed genes between regions such as zone 1 and zone 3 of the liver lobule to understand the metabolic functions and cell type distribution in each region (119). Scholars have also proposed that ST analysis can identify specific gene expression patterns related to liver diseases, which may help clinicians determine whether donor livers are suitable for transplantation (120). Recent spatial transcriptomics studies have demonstrated the ability to dissect intrahepatic immune and transcriptional heterogeneity in complex clinical settings. For instance, NanoString GeoMx digital spatial profiling was used to analyze liver biopsies from patients with chronic HBV and HDV or HIV co-infection, revealing infection-specific immune pathways, disrupted hepatocyte metabolism, and spatially distinct cellular patterns (121). However, the application of ST in clinical LT is currently limited. We only reviewed one study on ST in LT (46). In this study, researchers constructed a 70% hepatic ischemia-reperfusion model in mice and combined it with ST analysis to find that the central area of the liver lobule (zone 3) is the most sensitive to IRI. ST analysis showed significant differences in gene expression between different liver regions under homeostasis, with zone 3-specific differentially expressed genes enriched in metabolic pathways. After IRI, the related genes changed markedly in both injury and metabolic pathways. At the same time, IRI led to a decrease in the number and proportion of hepatocytes, while macrophages gathered in the severely damaged zone 3, with an increase in M1 type and a decrease in M2 type. Further experimental verification found that celastrol pretreatment could alleviate IRI in mice. Mechanistic studies suggested that celastrol may exert its effects by activating the HIF1α signaling pathway, thereby producing an ischemic preconditioning-like protective effect (**Figure 3**).

## 7 Current limitations in LT

Overall, the current single-cell omics technologies involved in LT are mainly focused on scRNA-seq, with a lack of further validation from other advanced omics. The vast majority of LT studies have employed case-control designs, and some have only established animal models of LT in mice. scRNA-seq has primarily been used for analyzing cellular heterogeneity in liver tissues and peripheral blood samples from LT recipients. Due to its high cell capture efficiency, the 10x Genomics platform is the most commonly used tool for scRNA-seq data analysis. Moreover, because of their relatively low cost and easy-to-follow workflows, flow cytometry and histological staining analyses are the most frequently used methods for validating research findings. In future omics studies related to LT, there is a need for more analytical methods involving multi-omics studies with mutual validation in longitudinal research. The application of ST in LT is relatively scarce, possibly due to the high complexity of data processing and analysis, as well as the high cost of ST technology, which thus limits its research and application in various fields (122). Furthermore, integrating multiple advanced omics such as scRNA-seq and spatial transcriptomics can link phenotypic information, cellular states, and spatial locations. In future omics research on LT, such integrative analyses can be further realized to provide more comprehensive insights.

## 8 Future directions in LT

The recent studies on scRNA-seq and ST in the field of LT have been outlined in the preceding text. Researchers have utilized scRNA-seq technology to analyze the heterogeneity of immune cell subpopulations after LT and have further identified specific cell subpopulations and injury mechanisms related to immune responses and IRI in LT. These findings provide potential molecular targets for the treatment of rejection reactions and IRI in liver transplantation, as well as new strategies for monitoring the immune function of recipients to enhance graft tolerance and improve transplant outcomes. While progress has been made in the field of LT, there are still limitations and gaps in the literature on LT research. Specifically, these limitations include the following points: 1) Insufficient patient sample sizes in clinical LT studies; 2) Lack of validation cohort studies; 3) Insufficient integration of multi-omics research; 4) Absence of time-series and longitudinal omics dynamic studies for long-term monitoring of LT-related cells and molecules; 5) The participation of advanced omics technologies such as single-cell assay for translocase accessible chromatin sequencing (scATAC-seq), single cell spatial transcriptomics and artificial intelligence (AI) technologies is insufficient; 6) The influence of genetic heterogeneity between donors and recipients on LT results was not fully considered. These limitations may affect the reference value of advanced omics in LT research. Rejection reactions remain one of the significant causes of LT failure, and IRI in LT can ultimately lead to acute liver dysfunction and even allograft rejection in recipients (93). There is an urgent need for advanced omics research to guide clinical LT to minimize graft damage to the greatest extent possible.

### 8.1 Increase the importance of sample size and validation

In all the clinical LT studies we reviewed, a common issue faced is the small sample size. Some studies (37, 40, 42, 43, 45) only included a few or even a single LT patient as the study cohort. However, studies with small sample sizes may produce significant biases due to the randomness of the samples and individual differences. Increasing the sample size can reduce such randomness, making the study results more representative and reliable. A larger sample size can enhance statistical power, enabling the study to more accurately detect significant differences and correlations (123). This is particularly important for the discovery of new biomarkers and therapeutic targets. Moreover, only four studies (35–38) validated the results of their study cohorts, with only one study (37) conducting validation experiments in LT patients, while the remaining three studies performed experimental validations in mouse LT models. Preliminary studies may have certain uncertainties in their conclusions due to factors such as small sample sizes and less rigorous study designs (123). In disease research, validation studies conducted at different times and places and on different sample populations can reduce the impact of these random errors on the study results, making the conclusions more reliable (124). Furthermore, all the clinical LT studies were single-center studies, and the clinical data obtained may have potential biases. In the future, multi-center studies may be needed to further validate the study results.

### 8.2 Integrated Multi-Omics Systematic Analysis of LT

Integrated multi-omics approaches have demonstrated great promise in LT research, especially in elucidating graft injury mechanisms and immune responses in preclinical models. Notably, our recent study highlighted how rodent LT models, combined with single-cell and spatial omics technologies, can uncover critical molecular pathways of ischemia-reperfusion injury and graft dysfunction, providing a foundation for translational validation and therapeutic discovery (125). Integrated multi-omics analysis combines data from various high-throughput technologies, providing a more comprehensive and in-depth understanding of cellular biology in both healthy and disease states. It enhances the quantity and accuracy of detected transcripts, offering valuable methods for better understanding molecular mechanisms and constructing predictive models (126–131). Two studies (38, 43) downloaded transcriptomic data from NCBI’s Gene Expression Omnibus (GEO) database and further statistically analyzed the obtained transcriptomic data through bulk RNA-seq, thereby validating the results obtained from scRNA-seq analysis. Another study (37) obtained transcriptomic data from peripheral blood samples of another independent cohort in the study and applied weighted gene co-expression network analysis (WGCNA) to bulk RNA-seq, yielding gene expression results consistent with scRNA-seq data. scRNA-seq provides high-resolution gene expression data at the single-cell level, revealing cellular heterogeneity and subpopulation characteristics. Bulk RNA-seq not only offers an overall gene expression profile of tissues but also further validates the research findings obtained from scRNA-seq analysis, providing an overall trend to ensure the comprehensiveness and reliability of the results. The combination of both allows for a comprehensive understanding of the immune microenvironment changes in LT from different levels. In further LT research, the joint application of technologies such as scRNA-seq and bulk RNA-seq for simultaneous transcriptomic data analysis of liver tissues and peripheral blood samples may help to obtain more comprehensive and reliable conclusions.

### 8.3 The necessity of dynamic and longitudinal omics studies in LT

Many clinical events, such as drug responses, may manifest differently at various stages throughout the disease progression. Dynamic omics studies may help uncover the causes and mechanisms underlying this developmental process (132, 133). In LT research, dynamic and longitudinal omics studies can capture certain biological changes in patients over time after transplantation. For example, in one of the studies we reviewed, Wang et al. (37) obtained different peripheral blood samples from a dynamic temporal and longitudinal study cohort and, through single-cell multi-omics analysis, discovered the longitudinal evolution of immune cells during LT recovery under immunosuppressive therapy. They also provided a four-stage framework for clinical LT, which aids in the clinical management of LT patients. Dynamic and longitudinal omics studies provide strong support for personalized medicine. By analyzing omics data from individuals at different time points, researchers can better understand an individual’s biological characteristics and their response to treatment. This, in turn, helps physicians develop more effective treatment plans or identify reliable drug targets, thereby improving treatment outcomes (134, 135). Longitudinal studies can frequently collect rich omics data from large cohorts, enabling researchers to capture the dynamic fluctuations of healthy states and conduct long-term monitoring. This is beneficial for identifying potential health risks and the disease progression process, thus providing a basis for early intervention (136). Overall, dynamic and longitudinal omics studies are necessary and valuable when long-term monitoring of certain drugs, cells, or molecules is required, as they can offer new insights into precise medical care and personalized management in clinical LT.

### 8.4 The prospects of advanced technologies in LT

scRNA-seq is primarily used to analyze gene expression profiles at the individual cell level; however, it does not provide information on gene regulatory elements, such as promoter or enhancer activities. In contrast, a single-cell assay for transposase-accessible chromatin using sequencing (scATAC-seq) enables the exploration of chromatin accessibility and gene regulatory dynamics at single-cell resolution. This technique employs Tn5 transposase to label genomic DNA in individual cells, specifically targeting open chromatin regions—those DNA regions not tightly wrapped by nucleosomes. The fragmented DNA is then sequenced to reveal chromatin accessibility landscapes (137). A study (138) utilized scATAC-seq to conduct a detailed analysis of ten human hematopoietic cell types, uncovering significant heterogeneity within common myeloid progenitors (CMPs) and granulocyte-monocyte progenitors (GMPs). Integration with scRNA-seq data further demonstrated that the activities of various transcription factors change dynamically across different stages of hematopoietic differentiation, with chromatin accessibility adjustments regulating the expression of key genes. This study provided new insights into the direct relationships between regulatory elements and their target genes, offering a novel perspective on the molecular mechanisms underlying hematopoietic lineage transitions. In another study (139), researchers combined scRNA-seq and scATAC-seq to investigate cellular states and functional changes in human pancreatic islets from patients with type 1 diabetes (T1D). Their findings elucidated the gene expression and regulatory mechanisms driving islet cell dysfunction in T1D, laying the groundwork for the development of potential therapeutic strategies. To date, scATAC-seq has not yet been applied in LT research. Future LT studies could attempt to integrate scRNA-seq and scATAC-seq as a multi-omics approach, enabling comprehensive analyses of the functional states of critical cell populations involved in the LT process. Such integration would allow for a more precise and thorough elucidation of immune cell activation mechanisms, as well as regulatory network dynamics and mechanisms underlying immune rejection and IRI.

Single-cell spatial transcriptomics combines the advantages of scRNA-seq and ST. Traditional transcriptomics can only provide the average gene expression levels of mixed-cell populations, while single-cell technologies can reveal cellular heterogeneity and the gene expression patterns of specific cell types. ST further provides the exact location of cells within tissues, thus enabling the study of cell-to-cell interactions and their functions in specific microenvironments (140, 141). During the process of collecting liver tissue samples for scRNA-seq analysis, the digestive nature of the liver requires washing the liver with heated collagenase solution, which may lead to the loss of gene expression of liver-resident immune cells or the removal of certain cell populations that may exist in the natural liver environment. Single-cell spatial transcriptomics can detect the complete immune cell expression of liver tissue regions without the liver digestion process, thus solving this problem (39). In some COVID-19 studies, researchers have generated single-cell/spatial organ maps from various types of patient samples by integrating scRNA-seq and ST data (142–145), with one study even identifying hepatocytes positive for SARS-CoV-2 RNA (145). In future LT research, the use of single-cell spatial transcriptomics can simultaneously understand heterogeneity at both the cellular and tissue levels, helping to discover certain important cells and molecular mechanisms in specific microenvironments. This provides more accurate cellular and molecular localization for the future discovery of potential therapeutic targets, and such specific targeted modifications will aid in more precise research and treatment.

With the continuous development of AI, it has been widely applied in many disciplines. Machine learning (ML) trains and learns from data through mathematical functions or rule sets and provides classification and predictive outputs with high accuracy (146). In the field of LT, there have been numerous studies on ML technologies (147). ML and deep learning (DL) models have been able to assess transplant candidacy and donor liver quality (148, 149), predict short-term and long-term survival rates of LT patients (150, 151), and can also be used to identify transplant rejection reactions and predict graft failure (152–156), among other applications. Studies have shown (157) that ML techniques can align scRNA-seq data with spatial data (such as spatial transcriptomics) to reconstruct genomic spatial patterns at the single-cell level. If ML technologies can be further combined with omics technologies and applied to clinical LT, it could greatly advance the progress of LT medicine.

### 8.5 Genetic heterogeneity and integration with single-cell omics

Genetic heterogeneity between donors and recipients is a critical factor influencing the success of LT. This heterogeneity not only involves well-known immune-related genes such as those within the major histocompatibility complex (MHC) but also encompasses numerous other genes that affect cellular function and immune responses. Ultimately, donor-recipient genetic differences can lead to varying LT outcomes, including immune rejection, IRI, differences in drug metabolism, and even disease recurrence (158, 159). By leveraging transcriptomic technologies, integrating genetic variation with single-cell transcriptomic maps offers a deeper understanding of the personalized mechanisms underlying LT success or failure. Early analytical approaches, such as LDSC-SEG, RolyPoly, and MAGMA, demonstrated that trait-associated signals could be enriched within specific cell or tissue types using bulk or single-cell datasets. However, these methods often fail to resolve intra-cellular heterogeneity and are not optimized for single-cell-level inference. In contrast, recently developed computational frameworks have significantly advanced the integration of genome-wide association studies (GWAS) with scRNA-seq and spatial transcriptomics data. Newer tools such as scPagwas, scBPS, scDRS, and gsMAP (specifically for spatial transcriptomics) enable higher-resolution insights by identifying genetically influenced cell subpopulations or spatial loci (160–163). These approaches facilitate a more precise dissection of the molecular mechanisms underlying liver transplant outcomes. For example, scPagwas allows researchers to prioritize key genes and pathways within genetically regulated subpopulations, potentially accelerating the development of targeted therapies (163). Furthermore, several studies have demonstrated the practicality of combining GWAS data with scRNA-seq to identify disease-associated cell types and states. For instance, Xiang et al. (164) integrated GWAS data with scRNA-seq to uncover hepatocyte subpopulations linked to primary biliary cholangitis. Another study (165) published in *Genome Medicine* utilized the integration of immune cell scRNA-seq and GWAS to identify critical immune cell types associated with severe COVID-19. In the context of LT, only through the joint analysis of genetic diversity and single-cell heterogeneity can researchers gain a deeper understanding of how genetic differences influence transplant outcomes. This integrated approach holds promise for harnessing genetic information to optimize transplant procedures, reduce the incidence of rejection, and ultimately improve long-term graft survival.

## 9 Summary

In summary, we have reviewed and summarized the research progress of some advanced omics technologies in LT in recent years, and assessed their value in clinical mechanism research and clinical translation. Advanced omics technologies, particularly scRNA-seq and ST, have significantly advanced our understanding of immune heterogeneity, injury mechanisms, and microenvironmental remodeling in LT. We have analyzed the current limitations and gaps in the application of advanced omics in LT research. These approaches have revealed dynamic changes in various immune cell populations and identified potential therapeutic targets, paving the way for precision medicine in the field of LT. Furthermore, the integration of multi-omics data, artificial intelligence (AI) algorithms, and dynamic longitudinal analyses is anticipated to deepen our insights into the complex biological processes underlying transplant outcomes. Finally, we have predicted the future directions of advanced omics technologies in clinical LT research, where specific targeted modifications will aid in more precise research and treatment.

## 10 Limitations

While this review provides a comprehensive overview of the current applications and future directions of advanced omics technologies in LT, several limitations must be acknowledged.

First, although we have summarized key studies utilizing scRNA-seq and ST, the available research in LT remains constrained by relatively small cohort sizes, predominantly single-center designs, and limited clinical validation. Second, most studies have focused on scRNA-seq, with insufficient exploration of other advanced omics technologies, such as scATAC-seq and single-cell spatial transcriptomics. Third, the integration of multi-omics data and dynamic longitudinal monitoring is still lacking, thereby restricting a comprehensive understanding of immune cell dynamics and microenvironmental changes over time after LT. Fourth, the incorporation of AI technologies, including machine learning and deep learning, remains limited in current LT-related omics research. Fifth, the genetic heterogeneity between donors and recipients—which significantly impacts transplant outcomes such as rejection, IRI, drug metabolism, and disease recurrence—has not been systematically addressed or integrated with single-cell omics analyses.

Addressing these limitations through larger, multi-center, longitudinal studies, combined with multi-omics integration and advanced computational methods, will be essential for promoting the clinical translation of omics technologies and advancing precision medicine in LT.
